# Utilization of Iodinated SpaceOAR Vue™ During Robotic Prostate Stereotactic Body Radiation Therapy (SBRT) to Identify the Rectal–Prostate Interface and Spare the Rectum: A Case Report

**DOI:** 10.3389/fonc.2020.607698

**Published:** 2021-01-07

**Authors:** Dylan Conroy, Kelly Becht, Matthew Forsthoefel, Abigail N. Pepin, Siyuan Lei, Abdul Rashid, Brian Timothy Collins, Jonathan W. Lischalk, Simeng Suy, Nima Aghdam, Ryan Andrew Hankins, Sean P. Collins

**Affiliations:** ^1^ Department of Radiation Medicine, Georgetown University Hospital, Washington, DC, United States; ^2^ Department of Urology, Georgetown University Hospital, Washington, DC, United States; ^3^ Georgetown University School of Medicine, Washington, DC, United States

**Keywords:** prostate cancer, stereotactic body radiation therapy, urethrogram, SpaceOAR Vue, magnetic resonance imaging

## Abstract

We describe the utilization of SpaceOAR Vue™, a new iodinated rectal spacer, during Robotic Stereotactic Body Radiation Therapy (SBRT) for a Prostate Cancer Patient with a contraindication to Magnetic Resonance Imaging. A 69-year-old Caucasian male presented with unfavorable intermediate risk prostate cancer and elected to undergo SBRT. His medical history was significant for atrial fibrillation on Rivaroxaban with a pacemaker. He was felt to be at increased risk of radiation proctitis following SBRT due to the inability to accurately contour the anterior rectal wall at the prostate apex without a treatment planning MRI and an increased risk of late rectal bleeding due to prescribed anticoagulants. In this case report, we discuss the technical aspects of appropriate placement and treatment planning for utilizing SpaceOAR Vue™ with Robotic SBRT.

## Background

The objective of treating prostate cancer with robotic stereotactic body radiation therapy (SBRT) is to treat the entire prostate and proximal 1-2 cm of the seminal vesicles. While doing so, it is crucial to limit radiation dose to the adjacent critical structures, most importantly the rectum. This is of particular importance in patients with underlying risks for rectal bleeding, such as those on anticoagulation or with inflammatory bowel disease (1, 2). Accurate and consistent identification of the rectum remains essentially important during treatment planning, as it is the dose-limiting critical structure. However, conventional CT scans lack the soft tissue resolution necessary for adequate visualization of the transition between the prostate and the rectum, especially at the prostatic apex (3). Reliance on CT imaging alone, therefore, risks delivering an inadequate dose of radiation to the prostate or an unacceptably increased dose to the anterior rectal wall. Coupled with the large radiation doses and steep dose gradients characteristic of SBRT, this inherent ambiguity in treatment planning leads to the potential for high rates of recurrence and/or rectal injury. Such rectal injury may be enhanced in patients on anticoagulants such as warfarin and/or clopidogrel, who are at high risk for delayed rectal bleeding (4).

One approach to reducing the rectal wall dose and thus minimizing GI toxicity is the use of a dissolvable, biocompatible hydrogel spacer placed in the perirectal space between the prostate and anterior rectum. In 2015, the SpaceOAR Hydrogel™ (Boston Scientific) received FDA approval following publication of a phase III trial which demonstrated a statistically significant reduction in both acute and late grade 1 rectal toxicities (5). Since then, the use of rectal hydrogel spacers has broadly increased with SBRT practice, and significant dose reductions to the anterior rectal wall during prostate SBRT are achieved when implementing perirectal spacers (6, 7).

This traditional spacer technology is clearly identifiable with non-invasive magnetic resonance (MR) imaging. Therefore, the standard approach to treatment planning uses MRI to visualize the soft tissue borders of adjacent critical structures, such as the rectum (8, 9). MRI has demonstrated superior definition of the prostatic borders and reduces the overall target volume by 30% when compared to CT imaging alone (10, 11). In addition, the interface between the posterior prostate and anterior rectum is better determined by MRI than by CT imaging, particularly with the visual aid of a rectal spacer and the anatomical separation it provides.

A dilemma arises, then, when patients with an absolute contraindication to MR imaging, such as presence of a non-compatible pacemaker (**Table 1**) present for radiation treatment (13, 14). Space-OAR Vue™ (Boston Scientific) is a novel synthetic, absorbable, iodinated cross-linked polyethylene glycol (PEG)-based hydrogel that is inserted transperineally to temporarily position the anterior rectum away from the prostate during radiation therapy. The intent of traditional perirectal spacers, as described above, is to reduce radiation dose to the anterior rectum and minimize gastrointestinal side effects of pelvic radiation. The traditional SpaceOAR™ perirectal spacer maintains this space throughout the course of prostate radiotherapy (for approximately 3 months) and is broken down by hydrolysis, completely absorbed by the patient, and excreted by renal filtration over time (after approximately 6 months) (15–17). SpaceOAR Vue™, through its iodinated cross-linked PEG, specifically introduces new technology by which radiopacity of the spacer for easy visualization on CT is achieved. The hydrogel is covalently bonded with iodine to ensure that there are never free-floating iodine molecules which could leave the mixture, meaning it is not contraindicated in patients with an iodinated contrast allergy. To our knowledge, no case reports on SpaceOAR Vue™-directed SBRT for prostate cancer have been published. Herein, we report our first experience utilizing SpaceOAR Vue™ during SBRT for clinically localized prostate cancer.

**Table 1:** Absolute and relative contraindications to MRI (12).

Absolute Contraindications	Relative Contraindications
Cardiac implantable electronic devices (e.g. pacemakers, implantable cardioverter defibrillators, cardiac resynchronization therapy devices)	Coronary/peripheral artery stents
Metallic intraocular foreign bodies	Programmable shunts
Implantable neurostimulators	Metal airway stents or tracheostomies
Cochlear implants	Intrauterine devices
Implantable drug infusion devices	Ocular prosthesis
Intravascular catheters with metal components (e.g. Swan-Ganz catheters)	Stapes implants
Metallic foreign bodies (e.g. bullets, shrapnel)	Surgical clips or wire sutures
Cerebral artery aneurysm clips	Joint replacements
Magnetic dental implants	Inferior vena cava (IVC) filters
	Harrington rods
	Tattoos less than 6 weeks old

## Case Presentation

A 69-year-old Caucasian male presented with an elevated PSA of 8.7 ng/mL. Digital rectal examination did not reveal palpable disease. A transrectal ultrasound-guided prostate biopsy demonstrated adenocarcinoma, Gleason’s grade 4 + 3 = 7, involving both lobes. Seven of the twelve sampled cores were involved, with up to 70% involvement. He was not an ideal surgical candidate due to an extensive cardiac history, including chronic atrial fibrillation on rivaroxaban, sick sinus syndrome requiring a dual-chamber pacemaker and biventricular implantable cardioverter-defibrillator (ICD), coronary artery disease, and ischemic cardiomyopathy. The patient was informed of his options for radiation therapy, including conventional fractionation, moderate hypofractionation, SBRT, and proton therapy. As the patient would be commuting a long distance to the treatment center, the convenience of a five-fraction treatment regimen was prioritized, and he elected for radiation therapy management with hypofractionated robotic SBRT. As he was unfavorable intermediate risk, he was counseled on the benefits and risks of ADT, and ultimately refused ADT due to undesirable side effects and concern of worsening his already poor cardiac health. Prior to treatment, gold fiducials and SpaceOAR Vue™ rectal spacers were placed without complication. Given the patient’s pacemaker and ICD, MRI was contraindicated and thus treatment planning with a CT scan with urethrogram was obtained. He was subsequently treated with SBRT, 36.25 Gy in 5 fractions.

## Methods

**Figure 1:**
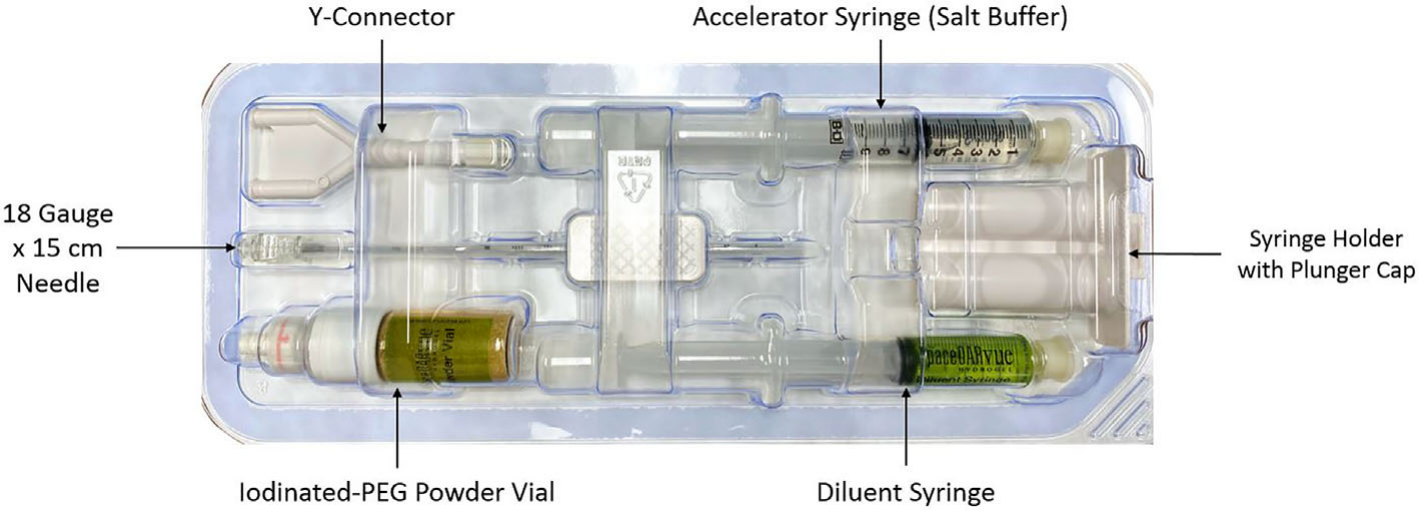
SpaceOAR Vue™ Assembly Kit.

CyberKnife^®^ (Accuray Incorporated, Sunnyvale, CA) treatment planning and delivery were conducted as previously published, with minor modifications (18, 19). Rivaroxaban was held for five days prior to fiducial/spacer placement per the patient’s Cardiologist’s recommendation to prevent significant bleeding. The patient was given an antibiotic for infectious prophylaxis. He was placed in the dorsal lithotomy position and prepped and draped in a standard manner. A transrectal probe was placed in the rectum to visualize the prostate. Six gold markers, followed by the rectal spacer, were placed into the prostate *via* a transperineal approach using a template and transrectal ultrasound guidance (19). The transperineal approach was utilized instead of the transrectal approach to minimize the risk of infection and assure coverage of the rectal prostate interface from the prostate base to apex. Six well separated (> 2 cm) gold markers were placed to maximize the accuracy of robotic tracking (20). Next, the grid was removed and attention was drawn to the placement of the spacer as previously comprehensively described (21). The components of the SpaceOAR Vue™ kit are shown in **Figure 1**. The needle tip of the 18 gauge needle was placed in the perirectal fat at mid gland. Hydrodissection to identify the tissue plane between the posterior prostate and anterior rectum (Denonvillier’s space) was performed. Utilizing both axial and sagittal views, care was taken to assure the space was midline and extended from the prostate base to apex (22). Finally, the spacer components were mixed and injected simultaneously over a ten second period. Of note, the pre-mixed components of SpaceOAR Vute™ are approximately twice as viscous as those of the original SpaceOAR, which leads to an increase in perceived resistance with injection of SpaceOAR Vue™, particularly in the last two to three seconds of injection. There were no acute complications following SpaceOAR Vue™ placement.

**Figure 2:**
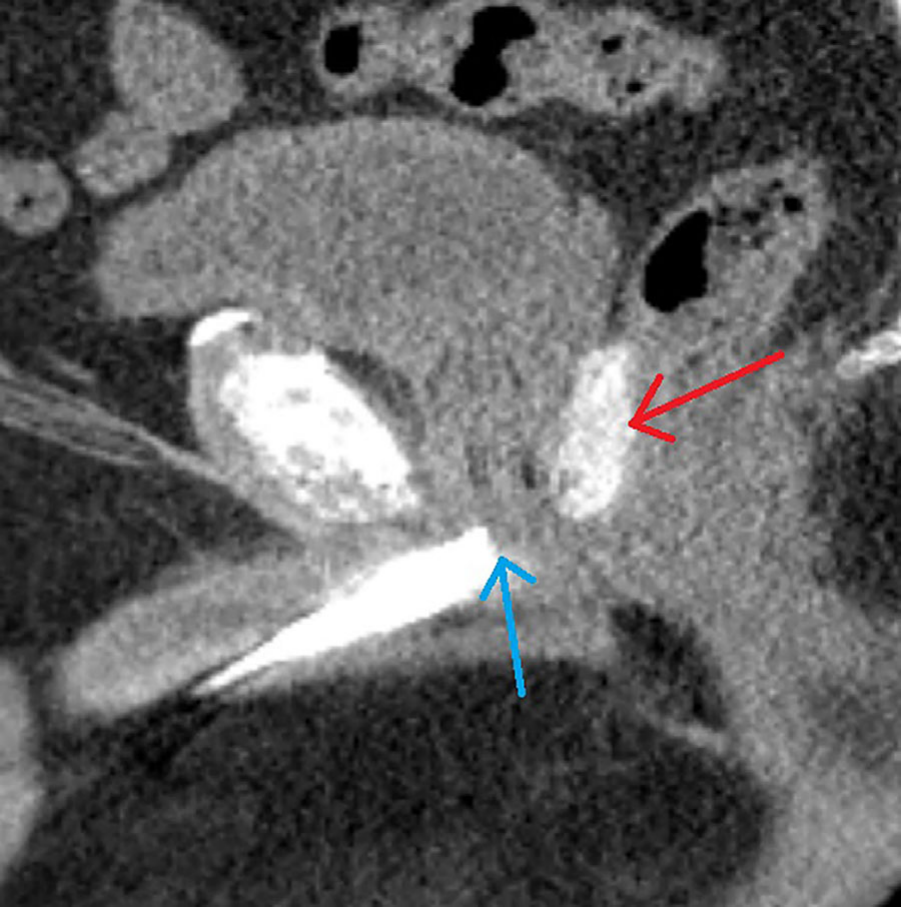
A 69-year-old male with intermediate risk prostate cancer had an implantable cardiac pacemaker which precluded magnetic resonance imaging for treatment planning. Thus he underwent placement of SpaceOAR Vue™ iodinated rectal spacer and a urethrogram CT image was obtained for treatment planning: Treatment planning sagittal computed tomography urethrogram images demonstrate the radiopaque spacer between the prostate and rectum (red arrow), as well as the beak of the urethrogram (blue arrow).

**Figure 3:**
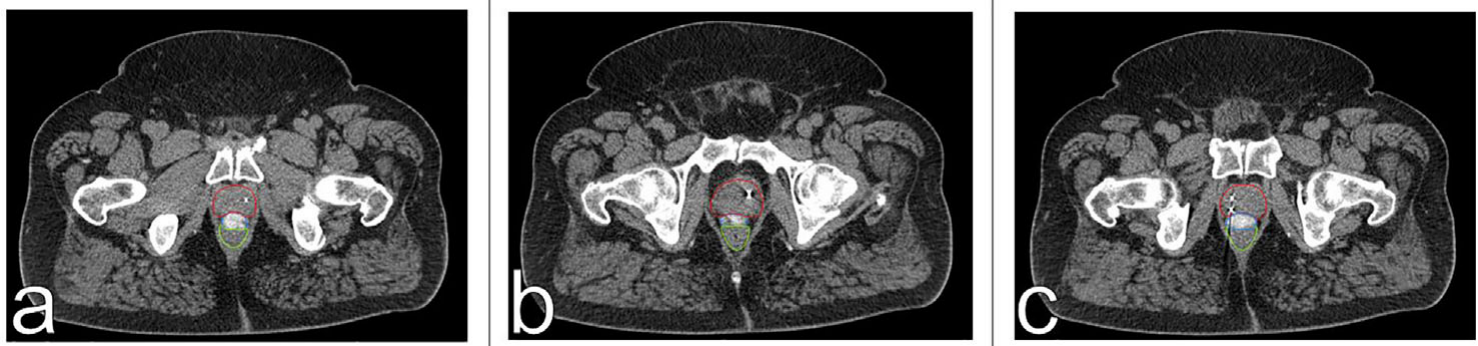
Axial CT images of spacer positioning at prostatic base **(A)**, mid-gland **(B)** and apex **(C)**. The spacer provides 1.1 cm **(A)**, 1.65 cm **(B)**, and 1.55 cm **(C)** of rectal separation. The prostate is contoured in red, the spacer in blue, and the rectum in green.

CT scan with a retrograde urethrogram for treatment planning was performed 7 days after fiducial/spacer implantation, allowing time for adequate fiducial fixation and resolution of procedure-associated tissue inflammation (23–26). Fused thin cut CT images (1.25 mm) were used for treatment planning. The iodinated spacer was clearly visible as a radio-opaque area between the prostate and rectum spanning from the prostate base to the apex (**Figure 2**). **Figure 3** shows contoured axial CT images of the symmetric spacer at the prostate mid-gland, base (1 cm superior to mid-gland) and apex (1 cm inferior to mid-gland) (22). The separation between the prostate and anterior rectal wall at these locations was 1.65 cm, 1.10 cm and 1.55 cm, respectively.

**Figure 4:**
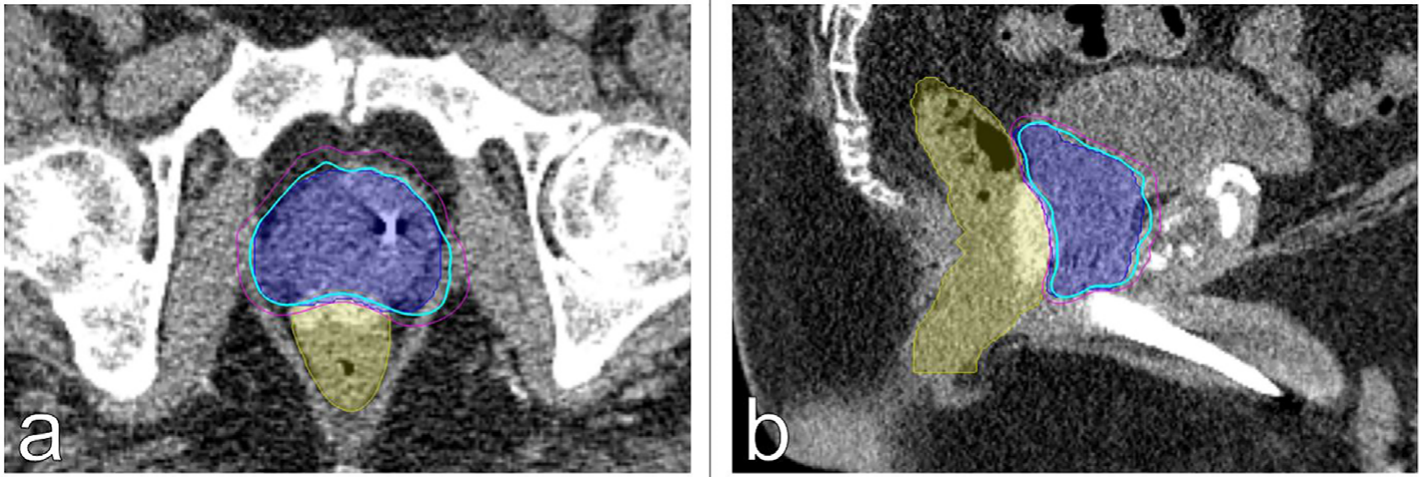
Treatment planning **(A)** axial and **(B)** sagittal computed tomography urethrogram images demonstrating the PTV (blue) and rectum with SpaceOAR Vue™ (yellow) are shown. Isodose lines shown as follows: 83% (blue), 75% (magenta).

**Figure 5:**
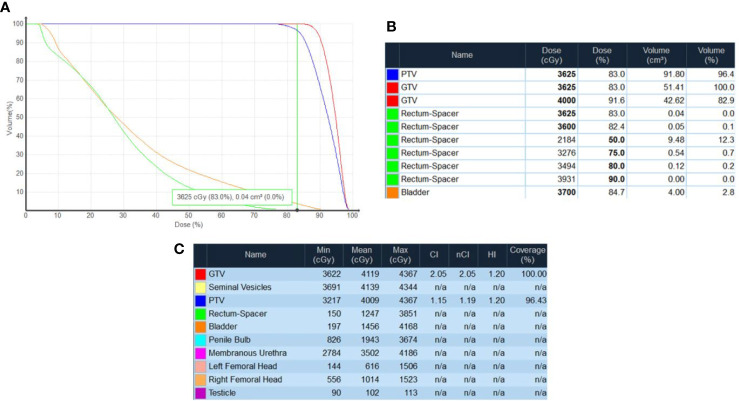
Dose-volume histogram (DVH) analysis using Multiplan (Accuray Inc., Sunnyvale, CA) inverse treatment planning. **(A)** Rectal DVH plot demonstrating V(36.25 Gy) = 0.05 cm^3^. **(B)** Rectal DVH (with spacer excluded from the volume of the rectum) demonstrating V(36 Gy) = 0.05 cm^3^ (0.1%). **(C)** Cumulative DVH demonstrating minimum, mean, and maximum doses delivered to OARs.

**Figure 6:**
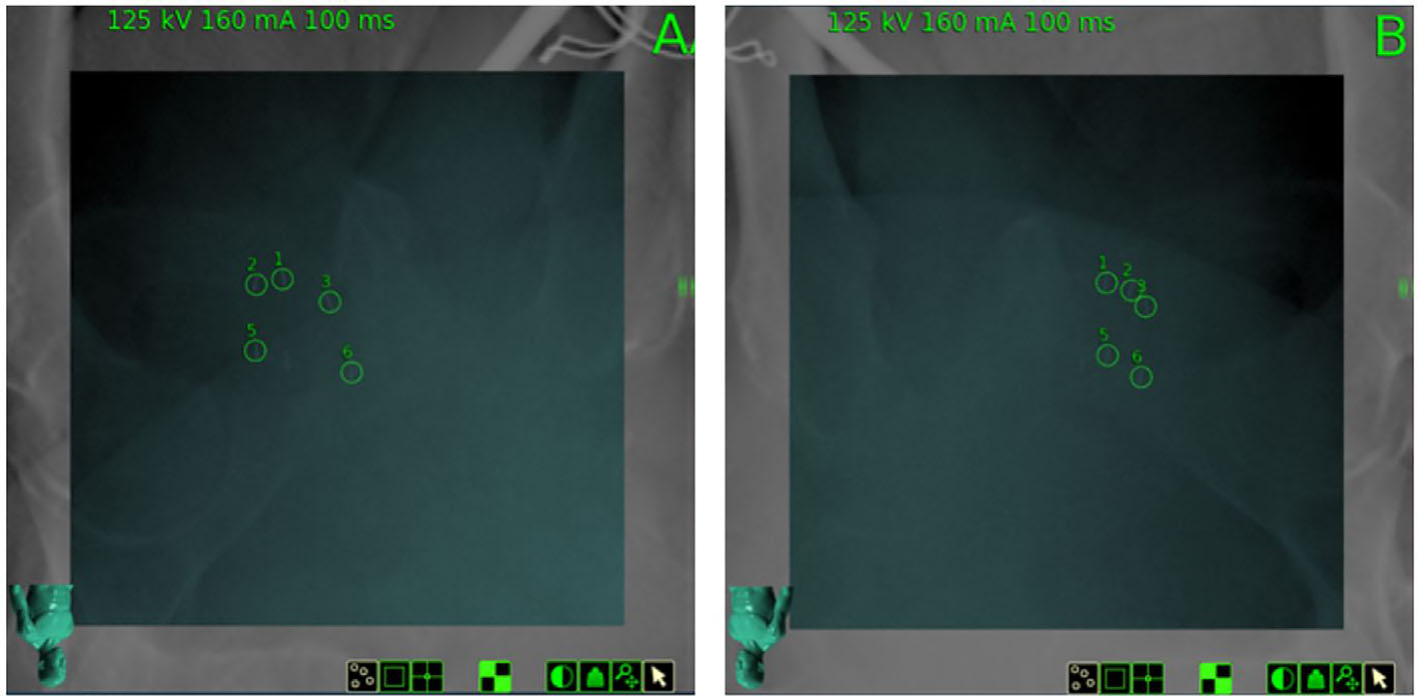
Digitally Reconstructed Radiographs (DRR) on day of treatment from planning CT and paired orthogonal X-Rays (Camera **A** and Camera **B**) obtained on the day of treatment, demonstrating gold fiducial alignment for 6D tracking. SpaceOAR Vue™ is not visible.

The clinical target volume (CTV), including the prostate and the proximal seminal vesicles, was created. The prostatic apex was defined utilizing the beak of the urethrogram and the rectal-prostatic interface was clearly identifiable with the presence of the iodinated spacer, particularly toward the prostatic apex (27, 28). The planning target volume (PTV) included a 3 mm (inferior, superior, and posterior) or 5 mm (anterior and lateral) expansion around the CTV. (**Figure 4**). The bladder, rectum, and membranous urethra were contoured and evaluated with dose-volume histogram (DVH) analysis during treatment planning using Multiplan (Accuray Inc., Sunnyvale, CA) inverse treatment planning technique as previously described (**Figure 5C**) (18, 29). For treatment delivery, the spacer was incorporated into the rectal volume to maximize rectal sparing as the dose response for rectal bleeding is unknown in patients on anticoagulants. For dosimetric purposes, the Hounsfield Units of the SpaceOAR Vue™ are manually set to 1 HU in order to prevent the computer algorithm from mistaking it for bone. Additionally, the spacer was then contoured separately from the rectum in order to calculate the true dose delivered to the rectum. No more than 1 cc of rectal volume was to receive 36 Gy. Assuming an α/β of 3 Gy for late bowel complications, this is biologically equivalent to approximately 74 Gy administered in 2 Gy fractions. Other rectal DVH constraints included the following: <40% rectal volume was to receive 50% of the prescribed dose, <20% to receive 80% of the dose, <10% to receive 90% of the dose, and <5% to receive 100% of the dose. The dose constraints were easily achieved in this scenario: V(36 Gy) = 0.05 cm^3^, V(50% Rx) = 20.5%, V(80%) = 3.1% (**Figures 5A, B**). The planning CT scan was also used to identify the gold fiducial markers and the image was converted into a digitally-reconstructed radiograph (DRR). Target position was identified multiple times with the utilization of these DRRs during each treatment using paired, orthogonal x-ray images (**Figure 6**) (30). The spacer was not visible on the DRR and did not interfere with prostate localization and beam targeting.

## Discussion

To our knowledge, this is the first report on the usage of SpaceOAR Vue™ rectal spacer in a patient undergoing SBRT for localized prostate cancer. The benefit of a rectal spacer is two-fold: it provides physical separation of the anterior rectal wall away from the prostate (and planned PTV) while also providing an easily identifiable boundary on MR imaging to aid in contouring. This last point is especially pronounced in demarcating the rectal-prostatic boundary at the apex of the prostate, where overdosing could damage the anterior rectum.

While late rectal/GI toxicity remains the dose-limiting toxicity of prostate radiation therapy, previous studies have demonstrated acceptably low late rectal toxicity (grade ≥ 2) with robotic SBRT in patients without a rectal spacer, on the order of 1.5% at 2 years to 4% at 7 years (31, 32). This is comparable and arguably superior to the cited rates of grade 2 and 3 rectal toxicity (15.6% and 7.0% at 5 years, respectively) with conventional fractionation (33). However, there was a noted higher incidence of late rectal bleeding in patients on anticoagulation therapy undergoing SBRT (31). Therefore, we felt comfortable recommending robotic SBRT for the patient described in this case report with the placement of a rectal hydrogel spacer.

In men with contraindications to MRI, such as our patient with an implantable pacemaker, treatment planning has previously been challenging due to the reliance on computed tomography imaging alone. As has been previously described, CT urethrogram imaging has been utilized to aid in the identification of the prostatic apex. In this prior study, 31 men with prostate cancer and contraindications to MRI were treated with urethrogram-directed SBRT. The 3-year incidence of ≥ Grade 2 GI toxicities was 9.7%. 19% of the study population were on anticoagulation, and the authors postulate that the increased incidence of GI toxicity could be attributed to this as well as other contributing comorbidities (34). This correlation between high comorbidity and increased risk of radiation therapy-related toxicity has been previously described (35). Additionally, the authors acknowledge the increased uncertainty in location of the anterior rectal wall with respect to the prostate when using urethrogram-based treatment planning without MRI fusion assistance (34).

The spatial separation provided by the traditional rectal spacers has already been clinically shown to lower the rates of acute rectal toxicity when compared with previous Linac SBRT reports performed without spacer placement (36). However, no studies have yet demonstrated the usage of this iodinated spacer as both a tool for planning and for reducing dose delivered to the rectum. For patients with contraindications to MRI and increased risk for late GI toxicity, we feel that the SpaceOAR Vue™ iodinated spacer would be of particular value and the ideal strategy to mitigate the risks of GI toxicity. As many practicing Radiation Oncologists and Urologists have become familiar and adept at placing the original SpaceOAR™ Hydrogel, with a cited successful placement rate nearing 99%, adoption of the SpaceOAR Vue™ system should be fairly seamless (37).

## Limitations

Without the MRI, it is difficult to know if there was intraprostatic injection or rectal wall infiltration during spacer placement (22). This places further importance on the technical skill and expertise of the physician placing the spacer, who must rely on real-time ultrasound imaging to confirm appropriate and safe hydrodissection and verify that the needle tip is properly within the perirectal fat and not the anterior rectal wall or prostatic capsule.

When compared to the traditional SpaceOAR™, SpaceOAR Vue™ is more expensive. This added expense is likely due the higher cost of covalently-bonding iodine to the polyethylene glycol gel. However, there is a consideration to be made for the potential cost savings of not undergoing an MRI and the dual-role of the SpaceOAR Vue™ as both rectal spacer and treatment planning tool.

## Conclusion

MRI will remain the preferred imaging modality to guide SBRT treatment for the time being, due to its currently unrivaled soft tissue resolution. However, contraindications to MRI should not preclude men with prostate cancer from access to the benefits of SBRT nor increase their risk of GI toxicity due to poor soft tissue differentiation between the prostate and anterior rectum. Utilization of iodinated rectal spacer in conjunction with urethrogram-directed SBRT is a safe and promising alternative to the planning and treatment of localized prostate cancer.

Additionally, the authors feel that SpaceOAR Vue™ would be beneficial over the traditional non-iodinated SpaceOAR™ in patients who undergo both a planning MRI and CT, as is standard practice. The contrast-enhancement of the SpaceOAR Vue™ on the planning CT scan would theoretically aid in accurate target delineation in the setting of an incongruent MRI, or in scenarios where the planning MRI and CT images do not fuse adequately or accurately.

The patient presented in this case will be followed life-long and we will be placing these iodinated rectal spacers in similar men with contraindications to MRI and following them similarly.

## Data Availability Statement

The datasets presented in this article are not readily available because data will not be made available for patient privacy concerns. Requests to access the datasets should be directed to SPC9@gunet.georgetown.edu.

## Ethics Statement

The patients/participants provided their written informed consent to participate in this study. Written informed consent was obtained from the individual(s) for the publication of any potentially identifiable images or data included in this article.

## Author Contributions

DC and KB were the lead authors, who participated in manuscript drafting, table/figure creation, literature review, and manuscript revision. MF aided in the figure creation. RH was responsible for the fiducial and spacer placement and aided in the review of the manuscript. SL and AR developed the SBRT treatment plans and contributed to the data analysis. AP, BC, JL, SS, and NA aided in the review of the manuscript. SC was the principal investigator who initially developed the concept of the study and the design, aided in the data collection, and drafted and revised the manuscript. All authors contributed to the article and approved the submitted version.

## Conflict of Interest

SC is a paid Speaker for Boston Scientific. SC and BC serve as clinical consultants to Accuray Inc. The Department of Radiation Medicine at Georgetown University Hospital receives a grant from Accuray to support a research coordinator.

The remaining authors declare that the research was conducted in the absence of any commercial or financial relationships that could be construed as a potential conflict of interest.

## Abbreviations

CT, computed tomography; CTV, clinical target volume; DRR, digitally-reconstructed radiograph; DVH, dose-volume histogram; GI, gastrointestinal; GTV, gross target volume; Gy, Gray; MR, magnetic resonance; PSA, prostate specific antigen; PTV, planning target volume, SBRT, stereotactic body radiation therapy.
